# Is the recent increase in cervical cancer in women aged 20–24 years in England a cause for concern?

**DOI:** 10.1016/j.ypmed.2017.12.002

**Published:** 2018-02

**Authors:** Alejandra Castanon, Peter Sasieni

**Affiliations:** a Queen Mary University, Wolfson Institute of Preventive Medicine, Charterhouse square, London EC1M 6BQ, United Kingdom; b King's College London, United Kingdom

**Keywords:** Uterine cervical neoplasms, Cervical screening, Cancer intelligence, Cancer rates, Early diagnosis, Overdiagnosis, Micro-invasion, Young women, Screen-detected, Cancer trends

## Abstract

The rates of cervical cancer (CxCa) in England among women aged 20–24 yrs increased from 2.7 in 2012 to 4.6 per 100,000 in 2014 (p = 0.0006). There was concern that the sudden increase was linked to the withdrawal of cervical screening in women aged 20–24 (a policy that affected women born since 1984).

We analyse granular data on age and FIGO stage at diagnosis using a generalised linear model to see whether the unprecedented increase in CxCa in young women in 2014 was linked to the change in 2012 to the age at which the first invitation to screening was sent (from 25.0 to 24.5).

Annual rates of CxCa per 100,000 women aged 20.0–24.5 yrs decreased gradually over time, whereas at age 24.5–25.0 yrs they increased from an average of 16 pre-2013 to 49 in 2015. An increase of 20.3 per 100,000 women aged 24.5–25.0 yrs (95% CI: 15.2–25.4) was associated with inviting women for screening at age 24.5 yrs instead of at age 25.0. At age 25.0–25.5 yrs, rates decreased by 23.7 per 100,000 after women were invited at age 24.5 yrs (p < 0.001). All these changes were limited to stage I CxCa. There was a dramatic increase in diagnoses at age 25 yrs in 2009–2011 associated with changing the age at first invitation from 20 yrs to 25 yrs. No changes were observed at age 26.0–27.0 yrs.

The increase in CxCa aged 20–24 is attributable to an increase in the proportion of women first screened aged 24.5 yrs. The increase was limited to stage I CxCa. There is no evidence of a lack of screening leading to increasing rates.

## Highlights

• There has been a dramatic increase in cervical cancer in women aged 20–24 in England.
• The increase in rates is limited to women aged 24.5–25.9 and to stage I cancers.
• The increase is attributed to a change in the age of first invitation for screening.
• Age at first invitation shifted from age 20 to 25.0 and more recently age 24.5.
• There is no evidence of a lack of screening leading to increasing rates.

## 1 Introduction

In 2004 the age of first cervical screening invitation in England was increased from 20 to 25 (Public Health England). This reflected a major change in screening policy and was based on evidence that screening at ages 20–24 provided no population benefit in terms of cancer prevention (Sasieni et al., 2003). In 2012, the age of sending out the first screening invitation was changed again; this time to 24.5 years. This was simply to enable women to be screened by their 25th birthday as it was noted that many women were only screened following a second reminder up to 6 months after the initial invitation (Health and Social Care Information Centre, 2013); it was not considered to be a major policy change. Approximately 62% of women aged 25–29 attend screening (Health and Social Care Information Centre, 2013). Amidst these changes HPV vaccination was introduced in 2008 for girls aged 12–13 with catch-up for those aged 14–18. Many people expected CxCa rates in women aged 20–24 to fall by 2014 as the vaccinated cohorts entered their twenties. However, in 2016 national statistics showed a worrying and substantial increase in the rate of cervical cancer (CxCa) at ages 20 to 24 (from 2.7 in 2012 to 4.6 per 100,000 in 2014, p = 0.006) (Office for National Statistics, 2016). Rates in this age group had been stable until 2012. Rates of CxCa in England at ages 25–29 have been steadily increasing from around the time of the first policy change onwards (from 10 per 100,000 in 2003 to over 20 per 100,000 in 2014).

The sudden increase in CxCa in women aged 20–24 sparked concerns that by withdrawing screening in 20–24 year olds, an epidemic stemming from lack of prevention was starting to emerge. This was particularly concerning as some argued that the lack of a reduction in rates showed that HPV immunisation had been ineffective. This paper describes an evaluation that was undertaken in response to these concerns. The analysis explores trends in CxCa in women aged < 30 yrs between 2006 and 2015, focussing on changes in the age at diagnosis and FIGO stage, over time. The aim was to elucidate what factors are driving the increase in cervical cancer among women aged 20–24 using routinely collected incidence data.

## 2 Methods

Notational convention: when we state the age in years without a decimal such as 26 we mean ≥ 26 and < 27. When we use an age range including a decimal we mean ≥ to the lower age and < the upper age. Thus “20–24” is the same as “20.0–25.0”.

### 2.1 Data

We plotted trends based on routinely published Cancer Registration Statistics for England from 1990 to 2015 (Office for National Statistics, 1990–2015); for Scotland from 1990 to 2014 from the Scottish Cancer Registry (Information Services Division. NHS National Services, 2016); for Wales data between 1990 and 2011 from the Welsh Cancer Intelligence and Surveillance Unit (The Welsh Cancer Intelligence and Surveillance Unit, 2013) and for cancers diagnosed between 2012 and 2015 from the Welsh Cancer Intelligence and Surveillance Unit website (Welsh Cancer Intelligence and Surveillance Unit, 2016). To ascertain the number of women invited for their first screening test at ages 20–24 we used data from Table 4 of the KC53 Part B returns published in the NHS Cervical Screening Programme Statistics, Source: KC53, Table 4 from 2010/11 to 2014/15 (Health and Social Care Information Centre and Screening and Immunisations, 2016).

In addition, by special request, we obtained (from the National Cancer Registration and Analysis Service, Public Health England) diagnoses of CxCa (ICD10 C53) in England by FIGO stage at diagnosis for ages 20–29 with detailed breakdown of those occurring at age 24.5–25.0, 25.0–25.5 and those at 25.5–26.0 for 2006 to 2015. These age ranges were chosen to examine in detail the effect of sending screening invitations six months earlier (i.e. at age 24.5 instead of at age 25.0).

It should be noted that the date of diagnosis recorded by the cancer registry should be the date on which the biopsy providing histological confirmation of the cancer was taken. HPV triage was introduced into cervical screening in England in 2012–2013. Prior to that a borderline (ASCUS) or mild (LSIL) smear would have been repeated at 6 months and women were only discharged from 6-monthly testing after three consecutive normal smears. Thus it could be 18 months or more from a screening abnormality until referral for colposcopy and cancer diagnosis. With HPV triage women should either be referred to colposcopy or returned to routine screening following their screen. National policy is for women to be informed of their results of screening within 2 weeks; in 2014–15 about 90% met the target (fewer than 1% took over 3 weeks) (Health and Social Care Information Centre, 2015). Time from referral to colposcopy appointment varied depending on the severity of cytology: 98% of high-grade cytology was offered an appointment within 4 weeks, but it took 8 weeks to include 98% of all referrals. Some clinics treat women with high-grade cytology at first colposcopy appointment, but most recall those with high-grade disease on biopsy for subsequent treatment. National data show that 82% all screen-detected cancers are diagnosed within four months of the screening test that led to colposcopy referral (Sasieni and Castanon, 2014).

### 2.2 Assumptions

The change in policy from first inviting women from age 20 to age 25 years was implemented in England over a 15-month period starting in August 2004. Since we did not have the date at which an individual was first invited for cervical screening, it is not clear whether women born between 26 August 1984 and 3 November 1985 were invited for screening before age 25. We estimate year of birth using year of diagnosis minus age (in years) at diagnosis. We categorised all women born before 1984 as ‘invited from age 20’, those born between 1986 and 1988 as ‘invited from age 25’. The change in policy inviting women at age 25.5 was implemented in December 2012 therefore we categorise women born from 1989 onwards as ‘invited from age 24.5’. Women born in 1984 and 1985 were labelled ‘mixed group’ because they may have either been invited from 20 or from 25 years.

We grouped FIGO stage as follows: Stage IA, Stage IB and Stage II or worse. Since some women had a recorded FIGO stage of I without further detail we proportionally distributed these cases between FIGO IA and FIGO IB. Those women with an unknown FIGO stage at diagnosis were distributed proportionally between stages IB and II or worse. We assume that those with missing stage were not stage IA because we know from previous research using the Audit of Invasive Cancer (Sasieni and Castanon, 2014) that women included in the Audit with un-staged cancers who eventually have their cancers staged rarely had stage IA cancer. We also performed a sensitivity analysis excluding cases of unknown FIGO stage.

### 2.3 Statistics

Cancer rates by nation (England, Scotland, Wales) and five-year age were smoothed against year of diagnosis using symmetric nearest neighbour smoothing (using the ‘running’ command in STATA).

An age-period-cohort analysis was not considered because i) we have little follow up for women invited from age 24.5, ii) we did not have number of cancers in six monthly intervals for all age groups and iii) not all cohorts reached age 30 by 2015. Instead a backward stepwise generalised linear model using the Poisson family and identity link function was fitted to obtain the change in rates associated with the age at screening invitation.

We include the following covariates in the regression: i) year of diagnosis; ii) a dummy variable (1/0) to indicate diagnoses in 2009, corresponding to the year in which reality TV star Jade Goody died. There were about half a million extra attendances to screening in England between her diagnosis (August 2008) and death (March 2009). At its peak, in March 2009 attendance was 70% higher than usual (Lancucki et al., 2012); iii) a variable to reflect the proportion of the birth cohort affected by the policy to screen from age 25.0 (X_25_ = 0 for birth cohorts < 1984, = 0.35 for 1984, = 0.70 for 1985 and = 1 for all other cohorts); iv) a variable to reflect the proportion of the birth cohort first invited for screening at age 24.5 (X_24_ = 0 for birth cohorts < 1989, = 0.75 for the 1989 cohort and = 1 for all other cohorts). Note that no woman has both X_24_ = 1 and X_25_ = 0_._ Sensitivity analyses on the effect of changing the proportion of women invited for screening in the ‘mixed’ cohorts were carried out. For the comparison of stage at diagnosis pre and post change in age at invitation we used a chi-square statistic with two degrees of freedom. p values of < 0.05 were considered statistically significant.

Data analyses were performed in STATA 13.1 (StataCorp. 1985–2013. Stata Statistical Software: Release 13.1. College Station, TX, USA: StataCorp LP).

## 3 Results

### 3.1 Comparison between countries with different policies

**Fig. 1:**
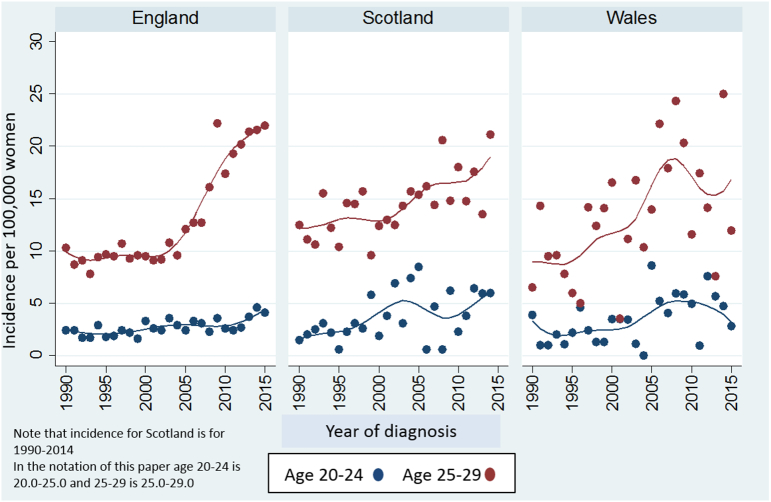
Cervical cancer incidence trends 1990–2015 by country in women aged 20 to 29 years.

Comparison of incidence rates in England (where screening women aged 20–24 yrs was phased out between 2004 and 2009) with rates in Scotland and Wales (where screening from age 20 yrs continued until after 2014) are presented in Fig. 1. Year-to-year variation in rates is much greater in Scotland and Wales than in England reflecting the very different population sizes.

In England an increase in rates among 20–24 year old women is evident from 2013 onwards. This coincides with an increase in the number of women under age 25 invited for screening for the first time (reflecting the change in policy to send out invitation 6 months earlier) from 35,435 invitations in 2010–11 to 189,544 in 2014–15, Table 1. Cervical cancer rates for women aged 20–24 yrs in Scotland and Wales have also increased over the last 10 years, Fig. 1. In fact, rates in Scotland in 2014 were as high as those in England. Rates at ages 25–29 yrs have increased in all three countries from around 2005 onwards; however those in England have seen the steepest rise.

**Table 1:** Number of women in England invited for screening for the first time in the year by age, 2010–15.

Age group at first invitation	Number of women receiving their first invitation to screening in England by financial year				
	2010/11	2011/12	2012/13	2013/14	2014/15
20–24	35,435	36,082	83,930	177,625	189,544
25–29	444,677	479,293	516,396	486,944	428,142
Total	480,112	515,375	600,326	664,569	617,686

### 3.2 Changes in the age at diagnosis of cervical cancer

**Fig. 2:**
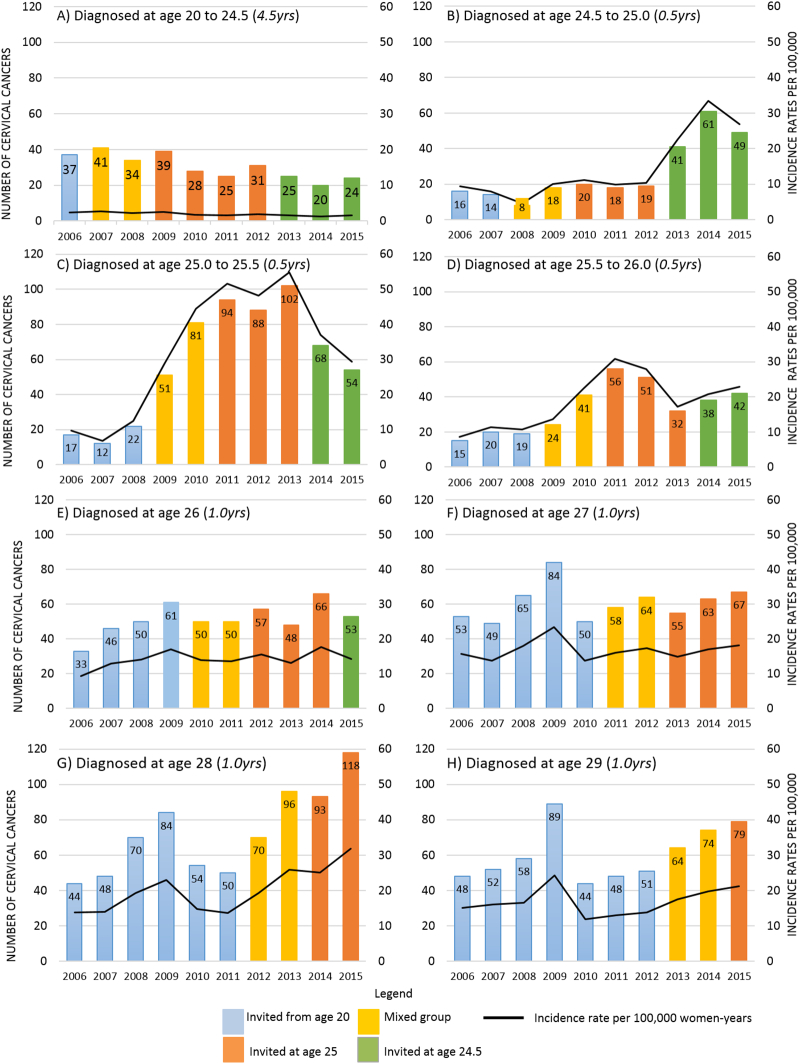
Numbers of cervical cancer (all stages) and incidence rates per 100,000 women-years in England at ages 20 to 29 years. Legend: Women invited to screening from age 20 yrs are shown in blue, women invited at age 25.0 yrs are shown in orange, women invited at age 24.5 yrs are shown in green and women for whom we did not know at which age they received their first invitation are shown in yellow. Black trend line show incidence rates per 100,000 women-years. (For interpretation of the references to colour in this figure legend, the reader is referred to the web version of this article.)

Numbers of diagnoses and incidence rates of CxCa diagnosed in England from 2006 to 2015 by age at diagnosis are presented in Fig. 2. The figure is colour-coded to indicate the age (based on birth cohort) at which women would have received their first invitation for screening. Details by age at diagnosis of the change in cancer incidence rates (increase or decrease) per 100,000 women-years and their association with calendar year, age at which women are first invited for screening and the Jade Goody effect are shown is Table 2.

**Table 2:** Results from the backward step generalised linear model: change in incidence rates per 100,000 women-years (95% confidence intervals) and p-values associated with age at first invitation for screening, calendar year and the Jade Goody effect by age at diagnosis.

Age at diagnosis	Change in incidence rates associated with calendar year, age at first invitation and the Jade Goody effect											
	Calendar year			Invited from age 24^a^			Invited from age 25^a^			Goody effect		
	Rate per 100,000	95% CI	p-Value	Rate per 100,000	95% CI	p-Value	Rate per 100,000	95% CI	p-Value	Rate per 100,000	95% CI	p-Value
20.0–24.5	‐ 0.08	(‐ 0.12 to ‐ 0.05)	**p < 0.001**									
24.5–25.0				20.3	(15.2 to 25.4)	**p < 0.001**						
25.0–25.5				‐ 23.7	(‐ 32.6 to ‐ 14.8)	**p p < 0.001**	43.7	(37.4 to 49.9)	**p < 0.001**			
25.5–26.0							14	(9.9 to 18.1)	**p < 0.001**			
26	0.4	(0.0 to 0.9)	p = 0.059									
27										7.3	(2.1 to 14.5)	**p = 0.006**
28							13.7	(9.8 to 17.6)	**p < 0.001**	7.9	(2.7 to 13.2)	**p = 0.003**
29							7.3	(3.1 to 11.4)	**p < 0.001**	9.9	(4.6 to 15.2)	**p < 0.001**

a Among the ‘mixed screening group’ we assume that 35% of women born in 1984 will have been invited at age 25 and 70% of those born in 1985. Further we assume that 75% of women born in 1989 were invited from age 24.5.

We observed a gradual decrease with calendar year in the rate per 100,000 women-years at age 20.0–24.5 yrs (annual decrease 0.08, 95% CI 0.05 to 0.12, p < 0.001); diagnoses decreased from 37 in 2006 to 24 in 2015, Fig. 2A. On the other hand the number of cancers diagnosed at ages 24.5–25.0 yrs increased suddenly from an average of 16 pre-2013 (when women were first invited either from age 20 or at age 25.0 yrs) to 49 in 2015 (when women were first invited at 24.5 yrs), Fig. 2B. The magnitude of the increase in rates at age 24.5–25.0 yrs associated with inviting women at age 24.5 yrs was 20.3 per 100,000 women-years (95% CI: 15.2 to 25.4, p < 0.001), Table 2.

An even more dramatic increase in diagnosis of CxCa was observed at age 25 yrs following the change in age at first invitation. Diagnoses increased four-fold between 2006–08 and 2010–12. The majority of the increase in this age group is observed among women aged 25.0–25.5 yrs between 2006 (n = 17) and 2013 (n = 102), after which the number diagnosed fell substantially (n = 54 in 2015) but are still much greater than when screening was from age 20, Fig. 2C. Inviting women at age 25.0 yrs was associated with an increase of 43.7 cancers per 100,000 women-years (95% CI: 37.4 to 49.9, p < 0.001), Table 2. Conversely the effect of inviting women for screening six months earlier (when they reach age 24.5 yrs) was to decrease incidence rates in women age 25.0–25.5 yrs by 23.7 per 100,000 women-years (95% CI: 14.8 to 32.6, p < 0.001) relative to what was observed when first invitation was sent at age 25.0 yrs; equivalent to a reduction in rates from about 51 to 27 per 100,000. However rates at age 25.0–25.5 are still high compared to 9.7 per 100,000 observed in 2006.

The increase in CxCa at age 25.5–26.0 yrs between 2006 and 2013 was more moderate: annual diagnoses increased from an average of 18 to 46, Fig. 2D. Inviting women to screening at age 24.5 yrs had no effect on incidence rates at age 25.5–26.0 yrs. However inviting women at age 25.0 yrs was associated with an increase in rates of 14 per 100,000 women-years (95% CI: 9.9 to 18.1, p = 0.001). At 26 yrs of age there was no significant effect of calendar year, age at first invitation, or Jade Goody on incidence rates (0.42, 95% CI: − 0.02 to 0.86, p = 0.059), Table 2 and Fig. 2E.

The increase in cervical screening in response to press coverage regarding Jade Goody's diagnosis and subsequent death was associated with a significant increase in the number of cancers diagnosed in 2009 at ages 27–29 yrs, Fig. 2G–H and Table 2.

Women first invited for screening at age 25.0 yrs had higher rates of diagnoses at ages 28.0–29.0 yrs (most likely reflecting screen-detected cancers diagnosed as a result of 3 yearly recall), Table 2. In particular, at age 28 yrs, an increase in cancers of 13.7 per 100,000 (95% CI: 9.8 to 17.6, p < 0.001) was associated with the cohort being first invited at age 25.0 yrs.

Results from the sensitivity analyses on the proportion of women invited at age 24.5 yrs (or 25.0 yrs) among women in the ‘mixed group’ did not significantly change the results (data not shown).

### 3.3 Changes in the FIGO stage at diagnosis

**Fig. 3:**
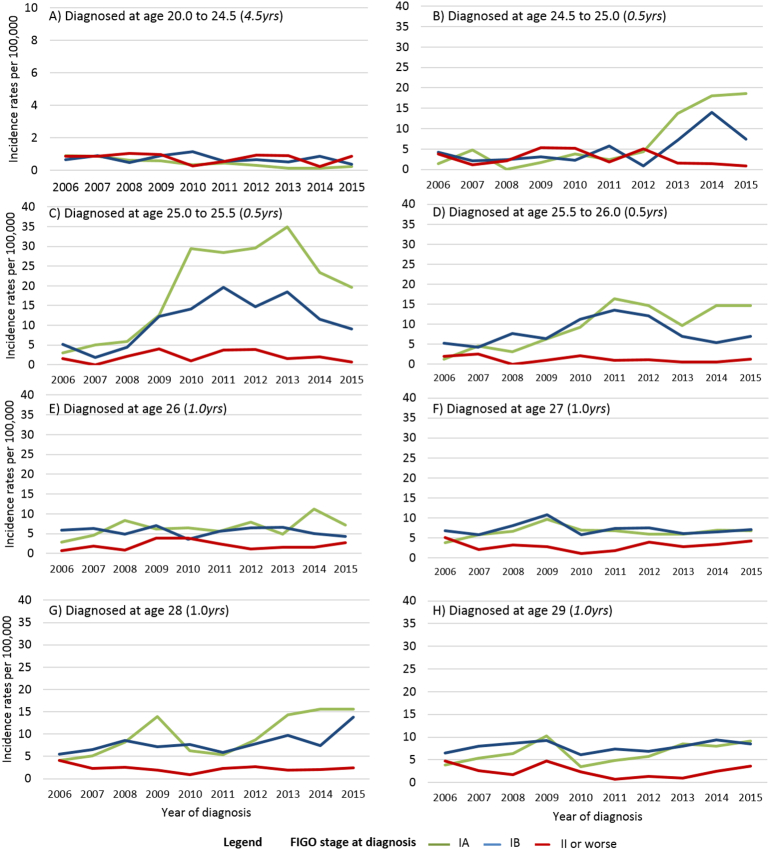
Incidence rates of cervical cancer per 100,000 women-years in England by age and FIGO stage at diagnosis. Legend: Green trend lines indicate rates of stage IA, blue trend lines indicate rates of stage IB and red trend lines rates of stages II or worse cervical cancer. Note that the scale on the Y axis in Panel A is different to the rest. (For interpretation of the references to colour in this figure legend, the reader is referred to the web version of this article.)

To understand the clinical significance of the increase in CxCa diagnosis we have looked at the FIGO stage at diagnosis among women aged < 30 yrs, Fig. 3 and Table 3. Results restricted to women with known FIGO stage are presented in Supplementary Table 1.

Following the ceasing of screening in women under age 25.0 yrs, the decrease in incidence rates with calendar time at age 20–24.5 was only statistically significant for stage IA cancers (p < 0.001). Among women aged 24.5–25.0 yrs the increase in rates per 100,000 women-years associated with inviting women at age 24.5 yrs was 15.5 (p < 0.001) for stage IA and 7.3 (p < 0.001) for stage IB. There were fewer stage II or worse cancers in 2013–2015 in this age group than in any year since 2007 (p = 0.001), Table 3.

**Table 3:** Results from the backward step generalised linear model: change in incidence rates per 100,000 women-years (95% confidence intervals) and p-values associated with age at first invitation for screening, calendar year and the Jade Goody effect by age and FIGO stage at diagnosis.

Age at diagnosis	FIGO stage	Change in incidence rates associated with calendar year, age at first invitation and the Jade Goody effect			
		Calendar year rates (95% CI), p-values	Invited from age 24^a^ rates (95% CI), p-values	Invited from age 25^a^ rates (95% CI), p-values	Jade Goody effect, rates(95% CI), p-values
20–24.5	IA	‐ 0.07 (‐ 0.10 to ‐ 0.04), **p** **<** **0.001**	−	−	−
	IB	‐ 0.03 (‐ 0.08 to 0.02), p = 0.268	−	−	−
	II +	‐ 0.03 (‐ 0.07 to 0.02), p = 0.241	−	−	−
24.5–25.0	IA	−	15.5 (11.6 to 19.4), **p** **<** **0.001**	−	−
	IB	−	7.3 (4.3 to 10.2), **p** **<** **0.001**	−	−
	II +	−	‐ 2.4 (‐ 3.9 to ‐ 1.0), **p** **=** **0.001**	−	−
25.0–25.5	IA	−	‐ 12.4 (‐ 19.4 to ‐ 5.4), **p** **=** **0.001**	27.7 (23.1 to 32.4), **p** **<** **0.001**	−
	IB	−	‐ 9.2 (‐ 14.2 to ‐ 4.1), **p** **<** **0.001**	14.2 (10.4 to 17.9), **p** **<** **0.001**	−
	II +	−	‐ 1.2 (‐ 2.8 to 0.5), p = 0.163	−	−
25.5–26.0	IA	−	−	10.8 (8.1 to 13.5), **p** **<** **0.001**	−
	IB	−	‐ 5.1 (‐ 8.9 to ‐ 1.2), **p** **=** **0.010**	5.2 (1.9 to 8.5), **p** **=** **0.002**	−
	II +	‐ 0.1 (‐ 0.3 to 0.1), p = 0.269	−	−	−
26	IA	0.5 (0.2 to 0.8), **p** **=** **0.002**	−	−	−
	IB	‐ 0.1 (‐ 0.4 to 0.2), p = 0.492	−	−	−
	II +	0.7 (0.22 to 1.1), **p** **=** **0.003**	−	‐ 4.0 (‐ 6.7 to ‐ 1.2), **p** **=** **0.004**	−
27	IA	−	−	−	3.5 (0.2 to 6.8), **p** **=** **0.040**
	IB	−	−	−	4.1 (0.5 to 7.6), **p** **=** **0.024**
	II +	‐ 0.6 (‐ 1.1 to ‐ 0.1), **p** **=** **0.018**	−	4.5 (1.4 to 7.7), **p** **=** **0.005**	−
28	IA	−	−	10.2 (7.4 to 12.9), **p** **<** **0.001**	8.1 (4.1 to 12.1), **p** **<** **0.001**
	IB	−	−	3.7 (1.3 to 6.1), **p** **=** **0.002**	−
	II +	‐ 0.1 (‐ 0.2 to 0.1), p = 0.331	−	−	−
29	IA	−	−	4.7 (1.9 to 7.4), **p** **=** **0.001**	5.2 (1.8 to 8.6), **p** **=** **0.003**
	IB	−	−	1.4 (‐ 1.3 to 4.2), p = 0.306	−
	II +	−	−	−	2.5 (0.2 to 4.8), **p** **=** **0.034**

The bold indicates p values that were statistically significant.

a Among the ‘mixed screening group’ 35% of women born in 1984 will have been invited at age 25 and 70% of those born in 1985. Further we assume that 75% of women born in 1989 were invited from age 24.5.

Among women aged 25.0–25.5 yrs, receiving an invitation at age 25.0 yrs (rather than from 20 yrs) led to an increase in stage IA incidence rates of 27.7 per 100,000 women-years (p < 0.001) and of 14.2 for stage IB cancers (p < 0.001). Inviting them six month earlier (at age 24.5 yrs) led to a more moderate subsequent decrease in stage IA (12.4, p = 0.001) and IB (9.2, p < 0.001) CxCa incidence rates. No evidence of a change in stage II or worse cancers (p = 0.163) in this age group was seen, Fig. 3C and Table 3.

At age 25.5–26.0 yrs the incidence rates of stage IA associated with inviting women at age 25.0 yrs increased by 10.8 per 100,000 women-years (p < 0.001). Rates of stage IB CxCa in women aged 25.5–26.0 yrs were similar in cohorts invited from aged 20 yrs and in cohorts invited at aged 24.5 yrs, Table 3.

After taking into account the gradual increase in incidence rates of stage II + cancer in women aged 26 yrs since 2006 (0.7 per year, 95% CI: 0.22 to 1.1, p = 0.003), inviting women at age 25.0 yrs resulted in a decrease in stage II + rates of 4.0 (1.2 to 6.7) per 100,000 women-years, Table 3.

An increase in stage IA and IB (but no increase in stage II +) CxCa was seen at age 27 yrs in 2009 (“Jade Goody effect”), Table 3. Although rates of stage II or worse CxCa at age 27 yrs have decreased with calendar time, cohorts first invited at age 25.0 yrs had an increase in stage II or worse incidence rates of 4.5 per 100,000 women (95% CI: 1.4 to 7.7, p = 0.005).

At age 28–29 yrs a significant increase in rates of stage IA CxCa associated both with the Jade Goody effect and first inviting women at age 25.0 yrs (so that the second invitation would be at age 28.0) was observed. An increase in rates of stage IB CxCa at age 28 yrs was associated with inviting women at age 25.0 yrs. At age 29 yrs there was an increase in stage II or worse cancer associated with the Jade Goody effect (2.5, 95% CI: 0.2 to 4.8 per 100,000).

In summary, the majority of the increase in CxCa since 2006 is due to an increase in stage IA and IB cancers associated with the change in screening policy whereby women are invited for screening at age 25.0 yrs (or 24.5 yrs) instead of from age 20 yrs. An increase in rates of stage II or worse CxCa associated with inviting women for screening at age 25.0 yrs was only observed among those aged 27 yrs and could be an artefact of multiple statistical testing, particularly given that rates of stage II + cancer decreased at age 26. Results among those with known FIGO stage (Supplementary Table 1) support these findings.

### 3.4 Comparison of cumulative risk of cervical cancer pre and post change in age at invitation

The cumulative number of cancers diagnosed at ages 24.5–27.0 yrs by birth cohort showed that inviting women at age 25.0 or 24.5 yrs (cohorts born from 1986 onwards) resulted in an average increase of 26 additional cancers per 100,000 women (over 3 years) of which 8 per 100,000 were stage IB and none were stage II or worse.

## 4 Discussion

An increase detection of prevalent cancer cases is expected when screening is first introduced. Results presented here show a change in diagnoses at age 24.5–25.5 of stage IA and IB CxCa associated with changes to the age at first invitation. At age 25.0–25.5 the increase associated with inviting women at age 25.0 instead of from age 20 (43.7 per 100,000 women–years) was greater than the decrease associated with sending invitations six month earlier (23.7 per 100,000). Reassuringly no increase in stage II or worse cancers was observed in women under age 27 yrs. In fact, numbers of stage II or worse cancers diagnosed at age 24.5–25.0 yrs in 2014 are lower than in any other year since 2007. The increase in stage II or worse CxCa at age 27 yrs and 29 yrs was unexpected and it is our intention to explore it in detail elsewhere. However, since the change in rates of stage II CxCa is not consistent across age groups we cannot rule out that the statistically ‘significant’ results are a spurious consequence of multiple-testing. The increase in diagnoses at age 28 yrs associated with inviting women at age 25.0 yrs was not anticipated but makes sense in that they are presumably screen-detected cancers from the second round of screening.

FIGO stage data were missing for between 16 and 20% of cases, we opted to impute them. This approach will have introduced uncertainty to the analyses and therefore we have been careful not to over interpret the results. Reassuringly, results restricted to those with known FIGO stage were similar. Unfortunately individual-level data on attendance to cervical screening and vaccination against HPV were not available.

If there was not a public demand to understand the recent increase in CxCa rates in women aged 20–24, we would have waited a further three years to allow all cohorts of women in this study the opportunity to reach age 30 by 2015 and hence be able to estimate cumulative incidence to age 30. However with the available data we have been able to show that the increase in incidence at age 20–24 is a direct result of inviting women for their first screening test at age 24.5 instead of at age 25 and not an epidemic stemming from lack of prevention.

There could be several operational reasons as to why the rates of cancer at age 25.0–25.5 in 2013 (54.9 per 100,000) were so much higher than those in 24.5–25.0 yr olds in 2014 (33.4 per 100,000). Since the policy is still to screen at age 25.0 (but with invitations sent out 6mths earlier) it may be that the time between invitation and screening is greater at 24.5 than it was at 25.0. It could also be that diagnoses following low-grade cytology in women screened at age 24.5 are not made until age 25.

Historically (2007–2012), 66% of CxCa at age 25.0 yrs and 24% at 26–29 yrs were diagnosed in women immediately following their first test (Castanon et al., 2013). Hence it is not surprising to see that most of the increase in diagnosis under age 30 yrs is stage IA cancers. That the majority (if not all) of the increase in rates under age 30 yrs is due to stage I cancer is encouraging since early diagnosis of CxCa increases the chances that treatment is both curative and fertility preserving. In England 93% of stage IA1, 88% of stage IA2 and 55% of stage IB1 cancers in women under age 30 yrs are treated conservatively by cone biopsy or trachelectomy (unpublished data from the Audit of Invasive Cervical Cancers) and five year survival for stage I CxCa is estimated to be 96% (Cancer Research UK, 2014).

Previous research (Landy et al., 2014) estimated that screening from age 25.0 yrs could lead to an increase of 14 cervical cancers per 100,000 women screened (compared with screening from age 20); based on an annual cervical intraepithelial neoplasia (CIN) grade 3 progression rate of 0.2% (age 20–24 yrs) to cancer. These excess 14 cancers are attributable to the failure to treat high-grade CIN prior to it becoming cancer at age 24.5 yrs. Here we estimate an increase of 33 cancers per 100,000 women screened (95 cancers in approximately 292,000 women aged 20.0–27.0 yrs screened each year). The additional 19 cancers per 100,000 women screened, over and above those 14 attributable to a failure to screen from age 20 yrs need understanding. The dramatic rise in IA cancer at ages 24.5–26.0 yrs and 28 yrs shown here cannot be solely attributable to early diagnosis at screening of cancers that would otherwise have been diagnosed a year or two later since we did not observe any commensurate fall at ages 26 and 27 yrs. Some of this rise will be explained by the increasing rates (as observed in Wales and Scotland) which are consistent with published literature (Foley et al., 2011, Health Protection Agency, 2010) suggesting greater (and earlier) exposure to HPV. Interestingly, preliminary results from Sweden (where no change in screening policy has occurred) also show an increase in stage IA CxCa rates in women under age 35 from 2012 to 2014 (Andrae et al., 2017).

Neither treatment of high-grade CIN following earlier screening, diagnosis at age 25 of cancers that would have been diagnosed age 26 or 27, nor the increase in underlying risk of HPV infection can fully explain the increase in rates observed in this study. We hypotheses that histology may have become more sensitive to micro-invasive cancers leading to diagnosis of slow-growing (or even regressive) disease at screening. It is possible, for instance, that more sections of excision biopsies are being examined for histologic diagnosis and consequently very small cancers are diagnosed that would otherwise have been recorded as high-grade CIN.

One may have expected to see a fall in cervical cancer rates resulting from HPV vaccination of women aged 14–18 yrs in 2008–2009. However, we consider it to be too early to draw conclusions regarding vaccine efficacy from CxCa incidence. It was only from March 2016 that women immunised (at age 17 or 18) as part of the first “vaccine catch-up” cohort attended screening at age 24.5. These women would have been aged 22–24 yrs in 2014 but, as we hypothesized previously, since very few women develop CxCa within 8 years of infection most women who get CxCa aged 20–24 yrs would have been infected before age 17 (Cuzick et al., 2010).

## 5 Conclusions

Screening from age 20 yrs, rather than from age 25 yrs, would not prevent any more cancers from spreading beyond the cervix (stage II or worse) by age 27 yrs. The substantial increase in stage I cervical cancers in 24 and 25 year old women, corresponds to changes whereby a high proportion of women are now screened for the first time between ages 24.5 and 25.5 yrs. Previously some of these early stage screen detected cancers would have been prevented by treatment of high-grade cervical intraepithelial neoplasia following earlier screening and a few would have been screen-detected later - at age 26 or 27 yrs. Others may be slow-growing cancers, some of which could be argued to be over-diagnosed.

The following is the supplementary data related to this article.

Supplementary Table 1 Women with known FIGO stage. Results from the backward step generalised linear model: change in incidence rates per 100,000 women-years (95% confidence intervals) and p-values associated with age at first invitation for screening, calendar year and the Jade Goody effect by age and FIGO stage at diagnosis. Supplementary Table 1

## Transparency document

Transparency document. Image 1
